# Association between Different Types of Tea Consumption and Risk of Gynecologic Cancer: A Meta-Analysis of Cohort Studies

**DOI:** 10.3390/nu15020403

**Published:** 2023-01-13

**Authors:** Fang Zheng, Kelie Chen, Jiamin Zhong, Song Tang, Sinan Xu, Weiguo Lu, Yihua Wu, Dajing Xia

**Affiliations:** 1 Department of Toxicology of School of Public Health and Department of Gynecologic Oncology of Women’s Hospital, Zhejiang University School of Medicine, Hangzhou 310058, China; 2 Department of Gynecologic Oncology of Women’s Hospital, Zhejiang University School of Medicine, Hangzhou 310058, China; 3 Research Unit of Intelligence Classification of Tumor Pathology and Precision Therapy, Chinese Academy of Medical Sciences (2019RU042), Hangzhou 310058, China; 4 Cancer Center, Zhejiang University, Hangzhou 310058, China

**Keywords:** tea consumption, incidence, gynecologic cancer, black tea

## Abstract

Plenty of studies have shown that tea has an effect of inhibiting gynecologic tumors. However, there still remained controversy of the association between tea and gynecologic tumors in epidemiological studies. In this study, PubMed, Embase, and Cochrane Database were used to search the literature from 1 January 1960 to 26 December 2022 to investigate the association between tea intake and gynecologic cancer risk. In total, 19 cohort studies with 2,020,980 subjects and 12,155 gynecological tumor cases were retrieved. The pooled relative risk (RR) of gynecologic tumor for tea intake was 1.00 (95% CI: 0.96–1.04). RRs were 0.94 (95% CI: 0.88–1.01) for ovarian cancer, 1.02 (95% CI: 0.97–1.07) for endometrial cancer, and 1.06 (95% CI: 0.91–1.23) for cervical cancer. Subgroup analyses were adopted based on the tea type and geographic location. Interestingly, significant preventive impact of non-herbal tea on ovarian cancer (pooled relative risk: 0.67; 95% CI: 0.55–0.81) was found, especially for black tea (pooled relative risk: 0.64; 95% CI: 0.51–0.80). Dose–response analysis indicated that although it is not statistically significant, a decreasing trend of ovarian cancer risk could be observed when the tea consumption was 1.40 to 3.12 cups/day. In conclusion, our findings suggested that ovarian cancer, but not other gynecologic cancers, could possibly be prevented by drinking non-herbal tea. In addition, the preventive impact of green tea on gynecologic cancer seemed to be relatively weak and needs further cohorts to validate it.

## 1. Introduction

Oncologic disease is a growing public health concern and presents a significant health care burden. For females, gynecologic cancers continue to be one of the most common and lethal cancers. Gynecological cancers involve several female genital organs, such as the ovary, the endometrium, the cervix, the vagina, the vulva, and the fallopian tubes. Cervical cancer and ovarian cancer were in the front rank in estimated cases and deaths worldwide [1]. Ovarian cancer represents the second most common malignant tumor in older women, especially in developed countries [2], whereas endometrial cancer has the sixth highest incidence of malignancy around the world [3]. Gynecologic cancer incidence was generally stable over time, with the exception of vulvar and endometrial cancers, which showed an increasing trend [4].

Along with the development of research, we could prevent most tumors with the knowledge in hand [5]. Numerous epidemiological studies have indicated that the risk and the mortality of cancer could be affected by diet [6,7]. However, it is difficult to yield consistent results with correlations between dietary habits and cancer incidences, possibly due to pathophysiological heterogeneity, differences in study durations, errors in dietary assessment, and the inverse time window of exposure during which dietary factors come into play [8].

Teas are widely consumed and very popular around the world, with potential health benefits [9]. Particularly, non-herbal tea, extracted from Camellia sinensis, is linked to a reduced incidence of tumor, as well as cardiovascular disorders [10]. Non-herbal tea can be divided mainly into green, black, white, oolong, Pu-erh, and yellow tea depending on the diverse fermentation method [11,12]. However, herbal teas such as ginseng are brewed from various parts of plant species such as stems and seeds, rather than the leaves, as in the case of Camellia sinensis. Compared to green teas, there is lower oxidation resistance in herbal teas [10]. Of note, the role of tea intake in carcinogenesis and cancer development is still controversial, especially among the different types of cancers [13,14]. The anti-gynecologic-cancer effects of tea, as well as its components, have been extensively studied. In ovarian cancer cells, tea may function to induce apoptosis [15], anti-angiogenesis [16], and adjuvant therapy [17,18]. Furthermore, a previous study has shown that tea has anti-angiogenic properties in endometrial cancer cells [19].

Regarding epidemiological studies, previous meta-analyses studying the relativity between tea intake and gynecological tumor incidence are conflicting. Some meta-analyses did not support the preventive impact of tea on ovarian cancer and endometrial cancer incidence [20,21,22]. In contrast, others found a significantly negative correlation between tea intake and gynecological tumor incidence [13,23,24]. These conflicts may be due to the differences in pathogenic factors and pathogenesis for diverse gynecological tumors and diverse study designs.

Hence, we sought for data from recent published cohort studies and undertook a meta-analysis to investigate the correlation between different types of tea and gynecological tumor incidence.

## 2. Materials and Methods

The meta-analysis has been registered in PROSPERO International Prospective Register of Systematic Reviews. The registration number was CRD42022304892. This study followed the Preferred Reporting Items for Systematic Reviews and Meta-analyses (PRISMA) statement [25].

### 2.1. Literature Search and Selection Criteria

PubMed, Embase, and Cochrane Library Database (from 1 January 1960 to 26 December 2022) were used to conduct the literature search without restriction. The keywords were shown as follows: (((ovarian OR ovary OR endometr* OR uter* OR cervical OR Gynecological OR CIN OR gynecologic OR gynecology OR vagina* OR vulva*) AND (malign* OR cancer* OR carcinoma* OR tumor* OR tumour*)) OR (ovarian cancer OR endometrial cancer OR cervical cancer OR vaginal cancer OR vulvar cancer)) AND tea. The Meta-analysis of Observational Studies in Epidemiology (MOOSE) guidelines was used to guide the design, execution, and reporting of our research [26].

After excluding duplication studies, two investigators (F.Z. and K.C.) independently performed the initial screening. Then, they reviewed the whole articles and references to find suitable studies to be included. There were four inclusion criteria, which were as follows: (1) only prospective cohort studies could be included; (2) the studies evaluated the association between teas and gynecologic tumors risk; (3) available relative risks (RRs) and 95% confidence intervals (CIs) or these parameters could be calculated; and (4) when the data from the same cohort were published twice or more, only the latest and most complete studies were included.

### 2.2. Data Collection and Quality Assessment

Two investigators (F.Z. and K.C.) extracted data of selected studies independently, and any differentials were settled by consensus or adjudicated by another investigator (J.Z.). The following information from each study was extracted: first author, publication year, country, study name, period, outcome, sample size, population characteristics, type of tea, time of follow-up, RR with adjustment, and corresponding 95% CI.

Two investigators (F.Z. and K.C.) independently evaluated the quality of each study using the NEWCASTLE-OTTAWA Scale (NOS) [27]. The total score ranged from 0 to 9 based on 3 different domains, including population domains, outcome assessment, and comparability of the groups. The quality classification was defined as follows: low quality (score < 5), medium quality (score 5–7), and high quality (score 8–9). The Grading of Recommendations Assessment, Development, and Evaluation (GRADE) system was applied to assess the certainty of evidence [28,29], and the overall evidence was defined as high, moderate, low, or very low.

### 2.3. Data Synthesis and Statistical Analysis

According to heterogeneity, a model of fixed-effect or random-effect was employed to pool the relative risks comparing the highest tea intake with the lowest categories, which were displayed in forest plots. A fixed-effect model was applied with a non-significant heterogeneity (*p* > 0.10 and *I*^2^ ≤ 50%). Then, the analysis was stratified by tea type and location. The publication biases were evaluated by Begg’s and Egger’s tests (*p* < 0.05 indicated significant), and a funnel plot was generated. Sensitivity analysis was performed by the leave-one-out method.

Dose–response relationship analysis was conducted by linear and non-linear methods. The intermediate dose was chosen to represent the exposure range. For the unbounded range, we estimated the highest range to be 1.5 times the lower bound and the lowest range to be zero. The person-years of different exposure gradient were not provided in 5 studies [30,31,32,33,34], and we estimated person-years by follow-up duration and subject number. Cups per day was regarded as the unit for dose–response analysis, and studies reporting total mass or other units were excluded from dose–response analysis [14,35,36]. All data analyses were conducted using STATA software (version 11.0; Stata Corporation, College Station, TX, USA). *p*-value < 0.05 indicated statistical significance.

## 3. Results

### 3.1. Study Selection Process and Inclusion of Studies

**Figure 1:**
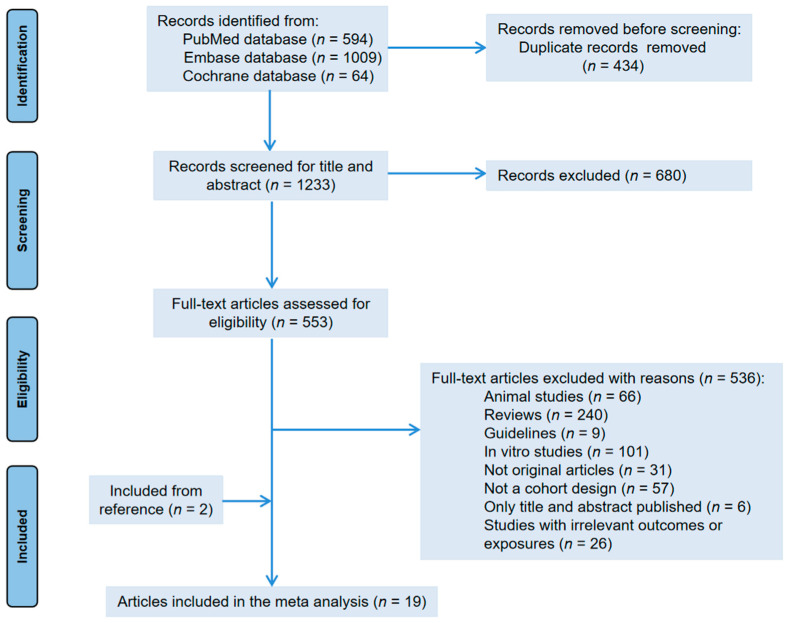
Flow chart of the study.

Figure 1 shows the flowchart of literature selection. We obtained 1667 articles from three electronic databases (594 from PubMed, 1009 from Embase, and 64 from Cochrane library database). After removing duplicate articles, we screened titles and abstracts based on inclusion and exclusion criteria. In addition, through reference collection and screening, 19 prospective cohorts were finally used for analyzation. Among them, 11 articles reported the correlation between tea intake and ovarian tumor risk [30,31,32,33,35,36,37,38,39,40,41]; 10 articles provided the incidence of endometrial cancer [30,32,33,34,35,42,43,44,45,46]); and 3 articles studied cervical cancer and tea drinking [14,30,47]. Due to the repetition of cohorts [30,40,41,45], we excluded data on endometrial cancer from Zheng et al. [30]. The data of Gates et al. [41] were used for dose–response analysis only. We did not find any prospective cohort studies evaluating the vaginal cancer incidence associated with tea consumption.

### 3.2. General Characteristics and Quality of the Studies

Table 1 presents the characteristics of 19 included prospective cohorts, published from 1996 to 2019. More details are shown in Table S1. Ten studies were performed in North America, six in Europe, and three in Asia. These studies involved 2,020,980 subjects and 12,155 gynecological tumor cases in total (3977 ovarian cancer cases, 6946 endometrial cancer cases, and 1232 cervical cancer cases). Follow-up time was distributed in the range of 7.5–26 years. In the quality assessment by NOS, only one study was of medium quality, and the others were of high quality (Table S2). Six studies clearly identified the type of tea or provided data on different types of tea and the incidence of gynecological tumors in subgroup analyses. Four studies described the broad categories of tea, such as herbal tea or non-herbal tea. Nine studies only counted the amount of tea consumed without distinguishing which type of tea. All except four studies included BMI as a covariate in multivariate-adjusted models [31,32,35,39]. Three of the studies included coffee intake as a covariate [31,37,46]. Through GRADE assessment, we found that the quality of the evidence was low, mainly because the study design was based on a cohort rather than a randomized controlled trial (Table S3).

### 3.3. Association between Tea Consumption and Gynecologic Cancer Incidence

**Figure 2:**
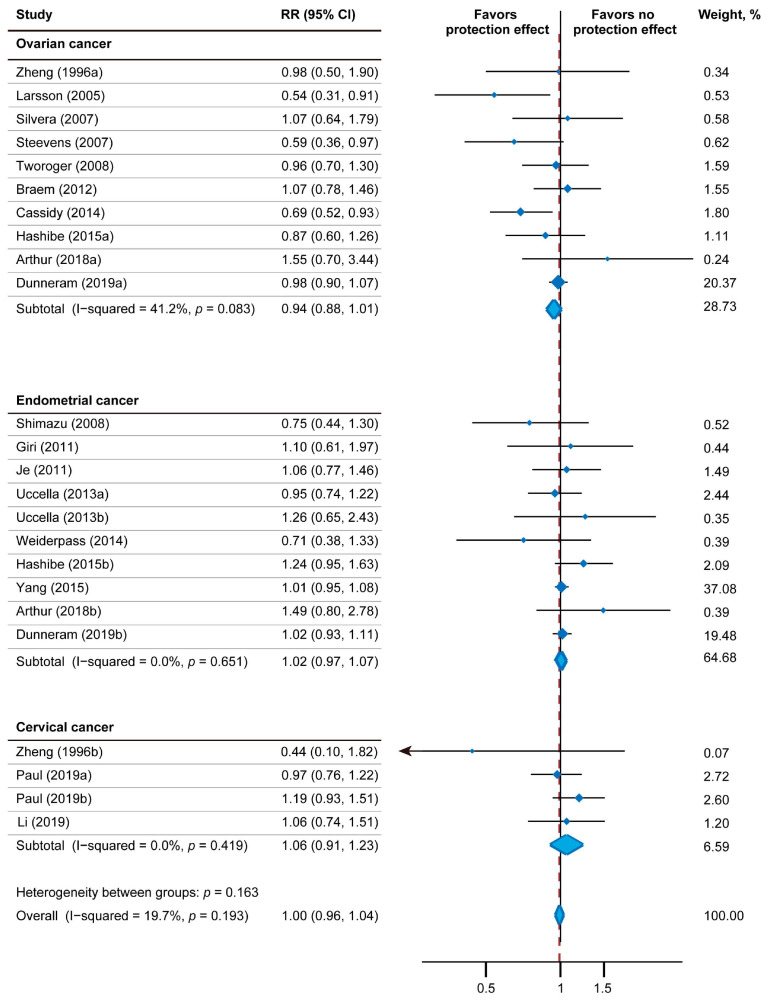
Forest plot of association between tea intake and gynecologic tumor risk. I-squared ≥ 50% represents substantial heterogeneity. RR—Relative risk; CI—Confidence intervals [14,30,31,32,33,34,35,36,37,38,39,40,42,43,44,45,46,47].

Figure 2 depicts the results of the relativity between tea consumption and gynecologic tumor incidence using a fixed-effect model, including 24 effect measures. The relative gynecologic tumor risk of tea intake was 1.00 (95% CI: 0.96–1.04). The statistical heterogeneity was low (*I*^2^ = 19.7%; P_heterogeneity_ = 0.193). Nine studies reported the impact of tea intake on endometrial tumor risk (pooled RR = 1.02; 95% CI: 0.97–1.07; *p* = 0.468; *I*^2^ = 0.0%; P_heterogeneity_ = 0.651; 918,339 participants; 6697 cases). Only three studies including 346,356 participants had analyzed the relationship between tea drinking and cervical tumor (pooled RR: 1.06; 95% CI: 0.91–1.23; *p* = 0.459; *I*^2^ = 0.0%; P_heterogeneity_ = 0.419). In regard to ovarian tumor risk, 10 studies including 917,121 participants and 3977 cases were included. Among them, three studies showed inverse association with statistical significance, four studies showed inverse association without statistical significance, and three studies showed positive association without statistical significance. Overall, the correlation between tea drinking and ovarian tumor incidence was not significant (pooled RR: 0.94; 95% CI: 0.88–1.01; *p* = 0.109; *I*^2^ = 41.2%; P_heterogeneity_ = 0.083).

Table 2 shows the outcome of subgroup analysis for tea consumption and different types of gynecological tumor incidence. Interestingly, subgroup analysis based on tea type suggested a significant impact of non-herbal tea on ovarian tumor risk (pooled RR: 0.67; 95% CI: 0.55–0.81; *p* < 0.001; *I*^2^ = 0.0%; P_heterogeneity_ = 0.706), especially the black tea (pooled RR: 0.64; 95% CI: 0.51–0.80; *p* < 0.001; *I*^2^ = 0.0%; P_heterogeneity_ = 0.690). However, we did not find any correlation between green tea and ovarian cancer due to data lacking from the prospective cohort.

### 3.4. Dose-Response Analysis

**Figure 3:**
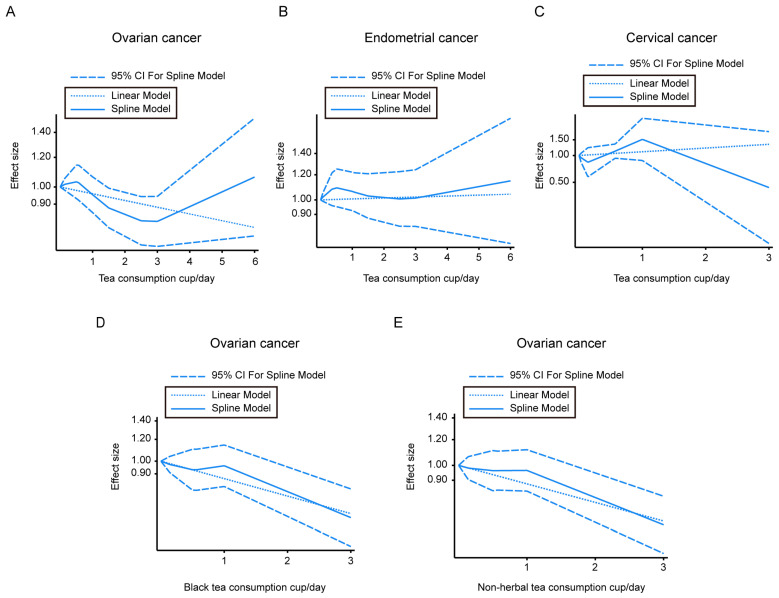
Dose–response correlation between different types of tea consumption and gynecologic cancer morbidity. (**A**–**C**) Dose–response analysis of tea intake and ovarian tumor morbidity (**A**), or endometrial tumor morbidity (**B**), or cervical tumor morbidity (**C**). (**D**) Dose–response analysis of black tea consumption and ovarian tumor morbidity. (**E**) Dose–response analysis of non-herbal tea consumption and ovarian tumor morbidity. CI—confidence intervals.

Seven cohorts were available for a dose-response analysis of the association of tea intake and the incidence of ovarian cancer, five studies were available for endometrial cancer risk, and three studies were available for cervical cancer risk. The U-shaped curve for ovarian tumor incidence associated with tea intake showed that although it is not statistically significant, a decreasing trend of ovarian tumor risk could be observed when the tea consumption was 1.40 to 3.12 cups/day. The linear model was also conducted and is shown in Figure 3A. The ovarian cancer incidence associated with black tea (Figure 3D) and non-herbal tea (Figure 3E) showed a dose-dependent decrease.

We did not find significant linear or nonlinear dose–response relations between tea and endometrial cancer incidence or cervical cancer risk (Figure 3B,C).

### 3.5. Sensitivity Analysis

Through leave-one-out analysis, most pooled RRs were kept stable in this meta-analysis, suggesting that no one study exerted a great influence on the pooled estimate (Figure S1). However, a sensitivity analysis after excluding Dunneram et al. [35] reported a slightly significant effect of tea on ovarian cancer. Considering the large population (35,372 subjects) and the recent long follow-up period (1995–2016, approximately 18 years) of the study, the protective effect against ovarian cancer of tea would be even stronger when these data were removed.

### 3.6. Publication Bias

**Figure 4:**
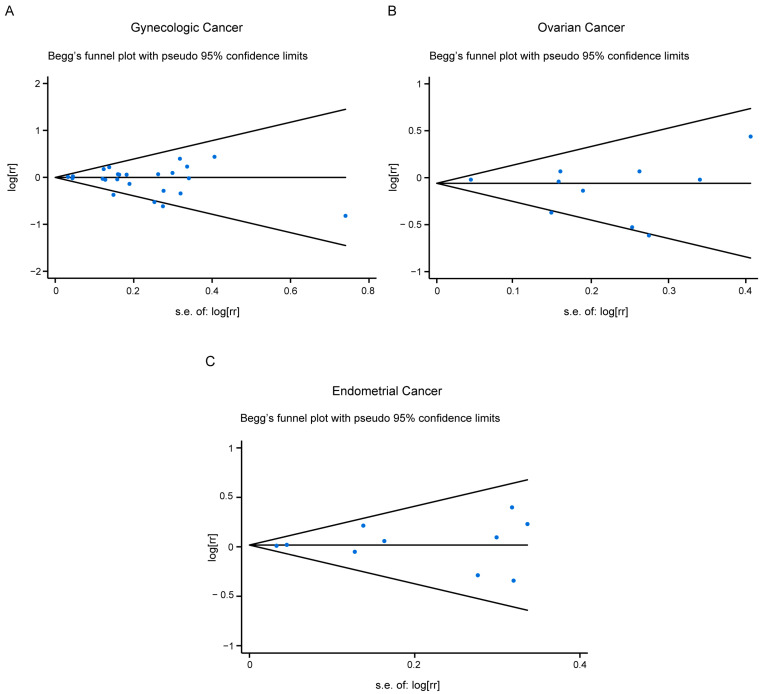
Publication bias for all studies included (**A**), or studies related with ovarian cancer risk (**B**), or endometrial cancer risk (**C**).

We found no publication bias in the included studies (Figure 4 and Table S4).

## 4. Discussion

In total, 19 cohort studies were included and analyzed in the present research, indicating no significant correlation between tea intake and the morbidity of overall gynecologic tumors or gynecologic tumors in different sites (ovary, endometrium, and cervix). Low statistical heterogeneity and small variation in the estimates in the sensitivity analysis suggest a stable association. Exclusion of studies that have a relatively large or small sample size did not influence the pooled RRs significantly, indicating that the sample size also did not affect the stability of our results. However, tea intake still has different effects on different types of gynecological tumors.

### 4.1. Association between Tea Intake and Ovarian Tumor

It is important to highlight that even though no consistency was observed in numerous studies of the anticancer effects of tea, and tea seems to exert a weak or no significant effect on most types of tumors [13,14,48], non-herbal tea, especially black tea, may have a preventive effect on ovarian tumor after separating tea type for subgroup analysis in our study, which was partially in line with the conclusions of Zhang et al. [23]. Their study indicated that both green tea and black tea had a significant impact on the prevention of ovarian cancer, and green tea was obviously better. However, this result should be taken with caution because their data were drawn from case–control studies mostly. Due to the lack of cohort studies related to green tea, our research did not analyze the role of green tea in the prevention of ovarian cancer or compare the difference in prevention effect. There are certain differences in the composition of tea due to distinct growing conditions, harvest time, and fermentation methods. Green tea is rich in catechins and caffeine, whereas black tea contains abundant theaflavins and thearubigins [48]. In previous studies, green tea has been observed to be more effective in preventing cancer than black tea, possibly owing to the difference in composition and the lower bioavailability of polyphenols in black tea [48]. However, with the increase of studies on black tea, it has been found that the antioxidant effect of theaflavins and thearubigins in black tea is nearly the same as green tea and even has a stronger down-regulation effect on oxidative stress and stronger antimutagenic activities [49,50,51,52]. Thus, more prospective cohort studies should be conducted to support the effect of green tea on preventing ovarian tumor. In addition, the conclusion that black tea prevents ovarian cancer needs to be further confirmed, because it may be easier to reach a positive conclusion based on observational studies and a relatively large sample size. After stratification by location, no significant effect was found, which may be due to the dominant effect of the type of tea.

One meta-analysis, including 11 case–control and 7 cohort studies, reported a significant negative relationship between tea drinking and ovarian tumor incidence (all study, RR = 0.86, 95%CI 0.76–0.96; case–control study, RR = 0.89, 95%CI 0.76–1.02; cohort study, RR = 0.80, 95%CI 0.62–0.97) [24]. Our study, which included more updated cohorts, showed that tea exerted no significant impact on ovarian tumor. This suggests that the ability of teas to prevent ovarian cancer may be weak or that only some types of tea (such as black tea) have the effect of reducing the incidence of ovarian tumor, whereas the impact of teas in preventing ovarian cancer is minimal.

In nonlinear dose–response analysis, there was a decreasing trend of ovarian cancer risk when the tea consumption was 1.40 to 3.12 cups/day. In this model, the role of tea in preventing ovarian cancer was not significant at higher consumption levels, which may be due to less data being available on higher tea consumption. It could also be that excessive tea consumption is less effective against tumors.

### 4.2. Association between Tea Intake and Endometrial Tumor

The present results regarding tea effect on risks of endometrial cancer are in line with the recently updated meta-analysis (RR = 1.06, 95% CI 0.96–1.18; P_heterogeneity_ = 0.027, *I*^2^ = 55.8%) of Zhang et al. [21]. In contrast with Zhang’s study, subgroup analysis (tea type and geographic location) was carried out in our study, and more complete cohort studies were included, with extremely low heterogeneity (P_heterogeneity_ = 0.651, *I*^2^ = 0.0%). Zhou et al. [53] reported that drinking green tea (RR = 0.78, 95% CI 0.66–0.92), but not black tea (RR = 0.99, 95% CI 0.79–1.23), may exert a protective impact on incidence of endometrial tumor, consistent with the research of Bulter et al. [54] in 2011. The difference may be partially due to their inclusion of both case–control studies and cohorts. As is generally acknowledged, case–control studies may enhance or amplify weak association effects [55,56,57]. On the other hand, differences in the classification of tea may affect the evaluation of the tumor-prevention contributions of different types of tea.

### 4.3. Association between Tea Intake and Cervical Tumor

As far as we know, this is the first meta-analysis to assess the association of tea intake and cervical tumor morbidity entirely through cohort studies. Our study found no association between tea intake and cervical tumor, including data from three different populations. This result was in line with our expectations because almost all cervical cancers resulted from high-risk subtypes of human papillomavirus (HPV) infection, and HPV screening and vaccination programs are effective preventive measures [58]. After adjusting for HPV status, the association was still not significant [47]. Notably, some vaginal cancers are also caused by high-risk types of HPV, and the correlation between vaginal cancer and tea consumption may also be weak.

### 4.4. Analysis of the Mechanism of Tea against Gynecological Tumors

Active ingredients of black tea such as flavonoids, phenolic acids, and methylxanthines contribute to many functions of black tea, including anti-tumor properties, age-defying properties, anti-inflammatory properties, and so on [59,60,61,62]. Studies have shown that the pharmacological benefits of black tea are mainly due to its polyphenols [63]. A large number of studies have shown that black tea can inhibit the occurrence and development of diverse tumors by regulating oxidative damage, endogenous antioxidants, mutagenic pathways, and transcription of the antioxidant gene pool [64]. In gynecologic tumors, consistent with our findings, the anti-cancer effect of tea seems to be more focused on ovarian cancer [65,66,67].

The results of our included studies on the prevention of gynecological tumors by green tea indicated that green tea could not significantly reduce the risk of endometrial and cervical tumor. Although the fact that green tea contains a similar component to black tea and has a similar effect on estrogen reduction [68,69] suggests that green tea may have anti-ovarian cancer effects, we still need to pay attention to the adverse effects of green tea on human health, as it contains more caffeine and EGCG than black tea [62]. In addition, according to studies in some Asian countries, tea contains some metal elements such as arsenic and aluminum, which may have adverse effects on human health [70,71]. Therefore, we can only assume with caution that green tea may have a role in the prevention of ovarian tumor. Therefore, more related prospective cohort studies are needed.

### 4.5. Advantages and Limitations

The current meta-analysis has some advantages that should be elucidated. First, a comprehensive and systematic search strategy was used to include all available cohort studies. Second, instead of combining data from all observational studies, our strength is that we included only prospective cohort data and could primarily prevent high bias that case–control studies can introduce, such as recall bias. Furthermore, because tea intake is not stable, this makes case–control studies even less credible. Third, the included cohort studies were all of high-quality except one of moderate quality, and this meta-analysis has a large sample size (2,020,980 subjects and 12,155 gynecological tumor cases). Moreover, the publication bias was not significant in our study. Sensitivity analysis also shows that the results are stable. It is important to highlight that we analyzed and compared the correlation between tea intake and the incidence of different gynecological tumors, which can more comprehensively reflect the similarities and differences of the preventive effect of tea on different gynecological tumors.

There also remain some limitations. First of all, although these studies had been adjusted for several risk factors for gynecologic cancer, there is still an uncontrolled possibility of confounders. Only three included studies adjusted for coffee consumption, and only four included cohorts adjusted for family history of cancer. Confounding factors including these may affect the accuracy of the results. Second, we excluded some cohorts due to incomplete publication data of some articles. Third, due to the lack of cohort study data, the association between tea and other gynecological tumors such as vaginal cancer is difficult to evaluate and analyze. Furthermore, histological types of gynecological tumors were not classified. Tumors such as ovarian cancer may have heterogeneity with different sensitivities and responsiveness to tea components, which is a major limiting factor for this research. Finally, different kinds of tea may exert distinct influences on the incidence of gynecological tumors, but only a few articles clearly stated the type of tea. Thus, we did not perform a correlation assessment for some types of tea due to the lack of available data. In addition, combining different types of tea for statistical analysis may be partly responsible for the weak overall association between tea intake and gynecological tumors.

## 5. Conclusions

In summary, the current meta-analysis provides the most updated and comprehensive study evaluating the correlation between tea and gynecological tumor. Our conclusions do not demonstrate a benefit of tea for the prevention of gynecological tumors. However, we observed that black tea may reduce ovarian cancer morbidity. Ovarian cancer is one of the most common cancers and the deadliest gynecologic cancer in women [72]. The present conclusion suggests that we can intervene in the occurrence and development of gynecologic tumor by exploring effective targets of black tea. In the past, we often thought that gynecological tumors were mostly related to estrogen. Based on this meta-analysis, black tea may have a preventive impact on ovarian cancer but had no significant correlation with endometrial cancer and cervical cancer. The diverse effects suggested to us that there are obvious differences in the mechanisms of the occurrence and development of different gynecologic cancers. More randomized controlled trials and large cohort studies including more areas and tea types are needed to reach more definitive conclusions.

## Figures and Tables

**Table 1:** Characteristics of the included studies.

Study	Country	Tea Intake	Type of Tea	Outcome	RR (95% CI)
Zheng (1996) [30]	USA	≥2 cups/day vs. never/monthly	non-herbal tea	Ovarian cancer	0.98 (0.50, 1.90)
				Cervical cancer	0.44 (0.10, 1.82)
Larsson (2005) [37]	Sweden	≥2 cups/day vs. never/seldom	black tea	Ovarian cancer	0.54 (0.31, 0.91)
Silvera (2007) [38]	Canada	≥4 cups/day vs. none	any tea	Ovarian cancer	1.07 (0.64, 1.79)
Steevens (2007) [39]	Netherlands	≥5 cups/day vs. <1 cup/day	black tea	Ovarian cancer	0.59 (0.36, 0.97)
Tworoger (2008) [40]	USA	2+ cups/day vs. ≤1 cup/week	any tea	Ovarian cancer	0.96 (0.70, 1.30)
Braem (2012) [36]	10 European countries	Quintile 5 vs. no take	any tea	Ovarian cancer	1.07 (0.78, 1.46)
Cassidy (2014) [31]	USA	>1/day vs. <1/week	black tea	Ovarian cancer	0.69 (0.52, 0.93)
Hashibe (2015) [32]	USA	≥1 cups/day vs. <1 cup/day	any tea	Ovarian cancer	0.87 (0.60, 1.26)
				Endometrial cancer	1.24 (0.95, 1.63)
Arthur (2018) [33]	Canada	>3 cups/day vs. none	any tea	Ovarian cancer	1.55 (0.70, 3.44)
				Endometrial cancer	1.49 (0.80, 2.78)
Dunneram (2019) [35]	UK	260 g/day vs. none	tea	Ovarian cancer Ovarian cancer	0.98 (0.90, 1.07)
			herbal tea		0.93 (0.75, 1.16)
			tea	Endometrial cancer Endometrial cancer	1.02 (0.93, 1.11)
			herbal tea		0.89 (0.71, 1.12)
Shimazu (2008) [42]	Japan	5 or more cups/day vs. ≤4 days/week	green tea	Endometrial cancer	0.75 (0.44, 1.30)
Giri (2011) [43]	USA	≥4 cups/day vs. non-daily	any tea	Endometrial cancer	1.10 (0.61, 1.97)
Je (2011) [44]	USA	2 cups/day vs. <1 cup/month	any tea	Endometrial cancer	1.06 (0.77, 1.46)
Uccella (2013) [45]	USA	5+ cups a week vs. never or once per month	non-herbal tea	Type Ⅰ endometrial cancer	0.95 (0.74, 1.22)
				Type Ⅱ endometrial cancer	1.26 (0.65, 2.43)
Weiderpass (2014) [34]	Sweden	>1 cup/day vs. 0 cup/day	black tea	Type Ⅰ endometrial cancer	0.71 (0.38, 1.33)
Yang (2015) [46]	UK	≥5 cups/day vs. <1 cup/day	any tea	Endometrial cancer	1.01 (0.95, 1.08)
Paul (2019) [47]	Singapore	tea drinkers vs. non-tea drinkers	green tea	Cervical cancer	0.97 (0.76, 1.22)
			black tea		1.19 (0.93, 1.51)
Li (2019) [14]	China	daily >4.0 g vs. less than weekly	any tea	Cervical cancer	1.06 (0.74, 1.51)
Gates (2007) [41]	USA	2+/day vs ≤1/week	non-herbal tea	Ovarian cancer	0.63 (0.40, 0.99)

Abbreviations: RR—Relative risk; CI—Confidence intervals.

**Table 2:** Subgroup analyses of association between tea consumption and gynecologic tumor risk.

Categories	Numbers of Studies	Numbers of Effect Measures	RR and 95% CI	*p* Value	*P* for Heterogeneity	*I*^2^ (%)
**Ovarian cancer**						
**Tea type**						
Non-herbal tea	5	5	0.67 (0.55, 0.81)	<0.001	0.706	0.0
Black tea	3	3	0.64 (0.51, 0.80)	<0.001	0.690	0.0
Herbal tea	1	1	0.93 (0.75, 1.16)	0.514	N/A	N/A
Tea (generally)	6	6	0.99 (0.91, 1.06)	0.704	0.837	0.0
**Geographic location**						
North America	6	6	0.88 (0.74, 1.06)	0.175	0.348	10.6
Europe	4	4	0.84 (0.64, 1.10)	0.196	0.032	65.8
**Endometrial cancer**						
**Tea type**						
Non-herbal tea	3	4	0.92 (0.75, 1.12)	0.394	0.544	0.0
Black tea	1	1	0.71 (0.38, 1.33)	0.284	N/A	N/A
Green tea	1	1	0.75 (0.44, 1.30)	0.298	N/A	N/A
Herbal tea	1	1	0.89 (0.71, 1.12)	0.316	N/A	N/A
Tea (generally)	6	6	1.02 (0.98, 1.08)	0.338	0.607	0.0
**Geographic location**						
North America	5	6	1.10 (0.95, 1.27)	0.196	0.669	0.0
Europe	3	3	1.01 (0.96, 1.07)	0.679	0.532	0.0
Asia	1	1	0.75 (0.44, 1.29)	0.298	N/A	N/A
**Cervical cancer**						
**Tea type**						
Non-herbal tea	2	3	1.06 (0.90, 1.25)	0.504	0.243	29.2
Black tea	1	1	1.19 (0.93, 1.52)	0.159	N/A	N/A
Green tea	1	1	0.97 (0.77, 1.23)	0.801	N/A	N/A
Tea (generally)	1	1	1.06 (0.74, 1.51)	0.749	N/A	N/A
**Geographic location**						
North America	1	1	0.44 (0.10, 1.88)	0.267	N/A	N/A
Asia	2	3	1.07 (0.92, 1.25)	0.389	0.496	0.0

## Data Availability

The data presented in this study are available in the inserted articles.

## Supplementary Materials

The following supporting information can be downloaded at: https://www.mdpi.com/article/10.3390/nu15020403/s1, Figure S1: Sensitivity analyses for studies related with ovarian cancer risk (a) or endometrial cancer risk (b) or cervical cancer risk (c) or all studies included (d) by leave-one-out method; Table S1: Characteristic of studies included in the meta-analysis; Table S2: Quality assessment; Table S3: Quality of evidence evaluated by GRADE approach; Table S4: Publication bias.
