# Supplementary material for: Association between Different Types of Tea Consumption and Risk of Gynecologic Cancer: A Meta-Analysis of Cohort Studies
Source: Nutrients. 2023 Jan 13;15(2):403. doi: 10.3390/nu15020403 (PMC9865679; doi:10.3390/nu15020403)
Supplement: Supplementary file 1 [file nutrients-15-00403-s001.zip › nutrients-2078865-supplementary.pdf]

Figure S1: Sensitivity analyses for studies related with ovarian cancer risk (a) or endometrial cancer risk (b) or cervical cancer risk (c) or all studies included (d) by leave-one-out method.

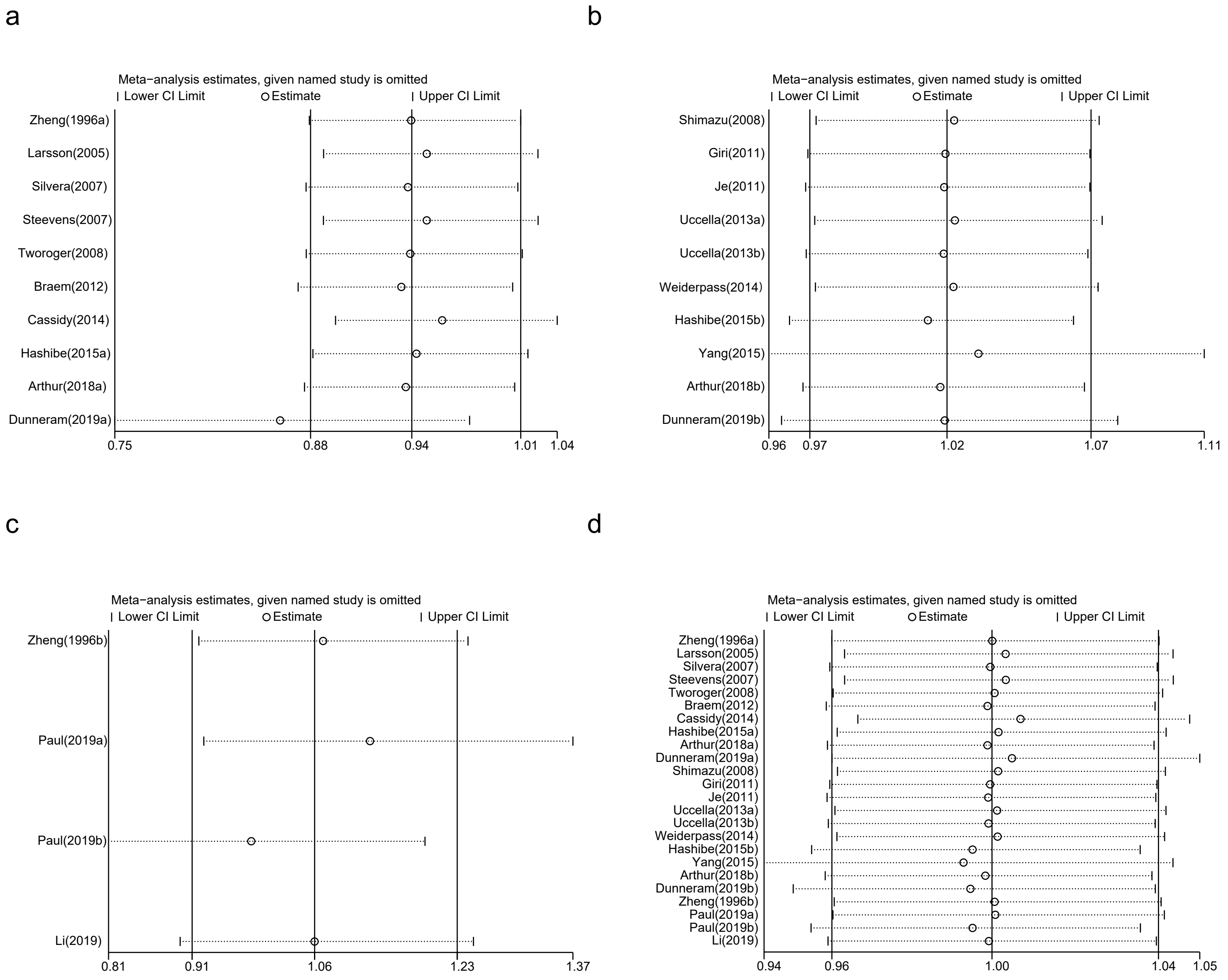

## Table S1: Characteristic of studies included in the meta-analysis

| Study | Study name | Period | Age/y | Quality[1] | Adjustments |
|---|---|---|---|---|---|
| Zheng (1996) [30] | Iowa Women's Health Study | 1986-1993 | 55-69 | 7 | Adjusted for age, education, smoking status, pack-years of smoking, physical activity, all fruit and vegetable Intake, waist/hip ratio, family history of cancer, age at menarche, age at menopause, age at first pregnancy. |
| Larsson (2005) [37] | Swedish Mammography Cohort | 1987-2004 | 40-76 | 9 | Adjusted for age in months; body mass index; education; parity; oral contraceptive use; intake of total energy; and consumption of fruit, vegetables, milk, liquor, beer, wine, and coffee. |
| Silvera (2007) [38] | National Breast Screening Study (NBSS) | 1980-2000 | 40-59 | 9 | Adjusted for age, smoking history, pack-years of smoking, alcohol intake, education, BMI in $kg/m^2$, parity, participation in vigorous physical activity, menopausal status, oral contraceptive use, energy intake, lactose intake, study center, and randomization group. |
| Steevens (2007) [39] | The Netherlands Cohort Study on Diet and Cancer | 1986-1999 | 55-69 | 9 | Adjusted for age, use of oral contraceptives, parity, cigarette smoking. |
| Tworoger (2008) [40] | Nurses' Health Study (NHS) | 1976-2004 | 30-55 | 8 | Adjusted for age, parity, oral contraceptive use, postmenopausal hormone use, tubal ligation, smoking status, and body mass index. |
| Braem (2012) [36] | European Prospective Investigation into Cancer and Nutrition (EPIC) | 1992-2011 | 25-70 | 9 | Stratified by center and age and adjusted for parity, oral contraceptive use, BMI, smoking status, alcohol consumption, total energy intake, duration of breastfeeding, menopausal status, height, and educational level. |
| Cassidy (2014) [31] | NHS and NHS II | NHS:1976-2010 NHS II:1989-2011 | NHS: 30-55 NHS II:25-42 | 9 | Stratified by age, calendar time, and cohort and adjusted for menopausal status, duration of oral contraceptive use, parity, history of tubal ligation, history of hysterectomy, duration of postmenopausal hormone use by type, family history of breast or ovarian cancer, quintiles of cumulative updated energy-adjusted lactose and caffeine intake, and quintiles of cumulative updated total energy intake. |
| Hashibe (2015) [32] | PLCO[2] cohort | 1992-2011 | 55-74 | 9 | Adjusted for age, sex, race, education, cigarette pack-years, and alcohol drinking frequency, smoking status, smoking frequency, smoking duration, time since stopping smoking for past smokers, and drinking frequency. |
| Arthur (2018) [33] | Canadian Study of Diet, Lifestyle, and Health (CSDLH) | 1992-2010 | 44-71 | 9 | Stratified by age at entry and adjusted for education, pack years of smoking, alcohol intake, total calories, BMI, physical activity, age at menarche, parity, breastfeeding, menopausal status, HRT use, oral contraceptive use, family history of breast cancer in a first degree relative. |
| Dunneram (2019) [35] | UK Women's Cohort Study | 1995-2016 | 35-69 | 9 | Adjusted for age, ethanol intake, duration of breastfeeding, physical activity, smoking, social class, menopausal status. Adjusted for age, ethanol intake, duration of breastfeeding, physical activity, smoking, social class, menopausal status, |

| | | | | | |
|---|---|---|---|---|---|
| | | | | | history of diabetes and history of hypertension. |
| Shimazu (2008) [42] | Japan Public Health Center-based Prospective Study | 1990-2005 | 40-69 | 9 | Adjusted for age, study area, body mass index, menopausal status, age at menopause for postmenopausal women, parity, use of exogenous female hormones, smoking status, green vegetable consumption, beef consumption, pork consumption.. |
| Giri (2011) [43] | WHI OS[3] | 1993-2005 | 50-79 | 8 | Adjusted for age, ethnicity, unopposed estrogen use, progestin + estrogen use, smoking and BMI. |
| Je (2011) [44] | NHS | 1980-2006 | 34-59 | 8 | Adjusted for age, BMI, age at menopause, age at menarche, parity and age at last birth, duration of oral contraceptive use, postmenopausal hormone use, pack-years of smoking, alcohol intake, and total energy intake. |
| Uccella (2013) [45] | Iowa Women's Health Study | 1986-2005 | 55-69 | 9 | Adjusted for age, diabetes, duration of HT use, hypertension, age at menarche, age at menopause, quartiles of body mass index, waist-to-hip ratio, smoking status, pack years of smoking, total energy and alcohol use. |
| Weiderpass (2014) [34] | Swedish Women's Lifestyle and Health cohort study | 1991-2009 | 30-49 | 9 | Adjusted for age, education, duration of hormonal contraceptive use, parity, duration of breastfeeding, smoking status and number of cigarettes/day, menopausal status, body mass index, and diabetes mellitus. |
| Yang (2015) [46] | UK Million Women Study | 1996-2011 | 58.5 ±4.6 | 9 | Adjusted for region, socioeconomic status, height, age at menarche, parity, duration of oral contraceptive use, age and status of menopause at study baseline, duration of hormone therapy for menopause, BMI, smoking, alcohol consumption, strenuous exercise, coffee consumption, and other nonalcoholic fluid intake. |
| Paul(2019) [47] | Singapore Chinese Health Study | 1993-2013 | 45-74 | 9 | Adjusted for age, dialect group, year of interview, level of education, smoking status, duration of oral contraceptive use, history of Pap-based test, parity, menopausal status, and daily total calorie intake. |
| Li(2019) [14] | China Kadoorie Biobank | 2004-2016 | 30-79 | 9 | Adjusted for education, occupation, marital status, household income, physical activity, intakes of red meat, fresh fruits and vegetables, body mass index, waist-hip ratio, family history of cancer, prevalent diabetes and menopausal status. |
| Gates(2007) [41] | NHS | 1984-2002 | 30-55 | 8 | Adjusted for age, duration of oral contraceptive use, parity, history of tubal ligation, smoking status, history of postmenopausal hormone use, physical activity, lactose intake, and total energy intake. |

1. Quality is assessed by NOS.
2. PLCO: Prostate, Lung, Colorectal, and Ovarian cancer Screening Trial.
3. WHI OS: Women's Health Initiative (WHI) Observational Study (OS).

**Table S2: Quality assessment**

| Study | Representativeness of the exposed cohort | Selection of the non exposed cohort | Ascertainment of exposure | Demonstration that outcome of interest was not present at start of study | Comparability of cohorts on the basis of the design or analysis | Assessment of outcome | Was follow-up long enough for outcomes to occur | Adequacy of follow up of cohorts | Total |
|---|---|---|---|---|---|---|---|---|---|
| Zheng (1996) [30] | 0 | 1 | 1 | 1 | 2 | 0 | 1 | 1 | 7 |
| Larsson (2005) [37] | 1 | 1 | 1 | 1 | 2 | 1 | 1 | 1 | 9 |
| Silvera (2007) [38] | 1 | 1 | 1 | 1 | 2 | 1 | 1 | 1 | 9 |
| Steevens (2007) [39] | 1 | 1 | 1 | 1 | 2 | 1 | 1 | 1 | 9 |
| Tworoger (2008) [40] | 0 | 1 | 1 | 1 | 2 | 1 | 1 | 1 | 8 |
| Braem (2012) [36] | 1 | 1 | 1 | 1 | 2 | 1 | 1 | 1 | 9 |
| Cassidy (2014) [31] | 1 | 1 | 1 | 1 | 2 | 1 | 1 | 1 | 9 |
| Hashibe (2015) [32] | 1 | 1 | 1 | 1 | 2 | 1 | 1 | 1 | 9 |
| Arthur (2018) [33] | 1 | 1 | 1 | 1 | 2 | 1 | 1 | 1 | 9 |
| Shimazu (2008) [42] | 1 | 1 | 1 | 1 | 2 | 1 | 1 | 1 | 9 |
| Giri (2011) [43] | 1 | 1 | 1 | 1 | 2 | 1 | 1 | 0 | 8 |
| Je (2011) [44] | 0 | 1 | 1 | 1 | 2 | 1 | 1 | 1 | 8 |
| Uccella (2013) [45] | 1 | 1 | 1 | 1 | 2 | 1 | 1 | 1 | 9 |
| Weiderpass (2014) [34] | 1 | 1 | 1 | 1 | 2 | 1 | 1 | 1 | 9 |
| Paul (2019) [47] | 1 | 1 | 1 | 1 | 2 | 1 | 1 | 1 | 9 |
| Li (2019) [14] | 1 | 1 | 1 | 1 | 2 | 1 | 1 | 1 | 9 |
| Dunneram (2019) [35] | 1 | 1 | 1 | 1 | 2 | 1 | 1 | 1 | 9 |
| Yang (2015) [46] | 1 | 1 | 1 | 1 | 2 | 1 | 1 | 1 | 9 |
| Gates (2007) [41] | 0 | 1 | 1 | 1 | 2 | 1 | 1 | 1 | 8 |

**Table S3. Quality of evidence evaluated by GRADE approach.**

| Grade criteria | Rating | Footnotes | Quality of the evidence |
|---|---|---|---|
| **Grade evidence profile on the association of tea consumption with risk of ovarian cancer** | | | |
| Study design | Low | Cohort studies | ⊕⊕○○ Low |
| Risk of Bias | 0 point | No serious | |
| Inconsistency | 0 point | $I^2$=41.2%, no considerable heterogeneity | |
| Indirectness | 0 Point | No serious | |
| Imprecision | 0 Point | No serious | |
| Publication bias | 0 Point | Undetected | |
| Large effect | 0 point | Pooled effect was 0.94 (95% CI: 0.90-1.01) | |
| Dose-response gradi | 0 point | No significant dose-response gradient | |
| **Grade evidence profile on the association of tea consumption with risk of endometrial cancer** | | | |
| Study design | Low | Cohort studies | ⊕⊕○○ Low |
| Risk of Bias | 0 point | No serious | |
| Inconsistency | 0 Point | $I^2$ = 0.0%, no considerable heterogeneity | |
| Indirectness | 0 Point | No serious | |
| Imprecision | 0 Point | No serious | |
| Publication bias | 0 point | Undetected | |
| Large effect | 0 point | Pooled effect was 1.02 (95% CI: 0.97-1.07) | |
| Dose-response gradi | 0 point | No significant dose-response gradient | |
| **Grade evidence profile on the association of tea consumption with risk of cervical cancer** | | | |
| Study design | Low | Cohort studies | ⊕⊕○○ Low |
| Risk of Bias | 0 point | No serious | |
| Inconsistency | 0 Point | $I^2$ = 0.0%, no considerable heterogeneity | |
| Indirectness | 0 Point | No serious | |
| Imprecision | 0 Point | No serious | |
| Publication bias | 0 point | Undetected and <10 studies included in meta analysis | |
| Large effect | 0 point | Pooled effect was 1.06 (95% CI: 0.96-1.04) | |
| Dose-response gradi | 0 point | No significant dose-response gradient | |

## Table S4: Publication bias

**Publication bias of all data**

Egger's test

| Std_Eff | Coef. | Std. Err. | t | P>\| t \| | 95% CI | |
|---|---|---|---|---|---|---|
| slope | .0198535 | . 0322117 | 0.62 | 0.544 | -.0469495 | .0866565 |
| bias | -.305526 | .3300415 | -0.93 | 0.365 | -.9899903 | .3789382 |

Begg's test

| | |
|---|---|
| adj. Kendall's Score (P-Q) | -30 |
| Std. Dev. of Score | 40.32 |
| Number of Studies | 24 |
| z | -0.74 |
| Pr ＞ \| z \| | 0.457 |
| z (continuity corrected) | 0.72 |
| Pr ＞ \| z \| (continuity corrected) | 0.472 |

**Publication bias of subgroup (ovarian cancer)**

Egger's test

| Std_Eff | Coef. | Std. Err. | t | P>\| t \| | 95% CI | |
|---|---|---|---|---|---|---|
| slope | -.0028468 | .0734813 | -0.04 | 0.970 | -.1722951 | .1666014 |
| bias | -.6400596 | .6252266 | -1.02 | 0.336 | -2.081835 | .8017154 |

Begg's test

| | |
|---|---|
| adj. Kendall's Score (P-Q) | -1 |
| Std. Dev. of Score | 11.18 |
| Number of Studies | 10 |
| z | -0.09 |
| Pr ＞ \| z \| | 0.929 |
| z (continuity corrected) | 0.00 |
| Pr ＞ \| z \| (continuity corrected) | 1.000 |

**Publication bias of subgroup (endometrial cancer)**

Egger's test

| Std_Eff | Coef. | Std. Err. | t | P>\| t \| | 95% CI | |
|---|---|---|---|---|---|---|
| slope | .0093312 | .0321267 | 0.29 | 0.779 | -.0647532 | .0834155 |
| bias | .1566356 | .4101066 | 0.38 | 0.712 | -.7890719 | 1.102343 |

Begg’s test

| | |
|---|---|
| adj. Kendall’s Score (P-Q) | 5 |
| Std. Dev. of Score | 11.18 |
| Number of Studies | 10 |
| z | 0.45 |
| Pr ＞ \| z \| | 0.655 |
| z (continuity corrected) | 0.36 |
| Pr ＞ \| z \| (continuity corrected) | 0.721 |
